# Custo-efetividade de lanches e refeições na alimentação escolar de um município do Sul do Brasil

**DOI:** 10.1590/0102-311XPT199024

**Published:** 2025-09-19

**Authors:** Gabriela Marques Costa, Julia de Souza Iparraguirre, Flávia Mori Sarti, Daniele Botelho Vinholes

**Affiliations:** 1 Universidade Federal de Ciências da Saúde de Porto Alegre, Porto Alegre, Brasil.; 2 Escola de Artes, Ciências e Humanidades, Universidade de São Paulo, São Paulo, Brasil.

**Keywords:** Planejamento de Cardápio, Serviços de Alimentação, Programas e Políticas de Nutrição e Alimentação, Análise de Custo-Efetividade, Alimentação Escolar, Menu Planning, Food Services, Nutrition Programs and Policies, Cost-Effectiveness Analysis, School Feeding, Planificación de Menú, Servicios de Alimentación, Programas y Políticas de Nutrición y Alimentación, Análisis de Coste-Efectividad, Alimentación Escolar

## Abstract

Diante da importância da efetiva execução do Programa Nacional de Alimentação Escolar na promoção da segurança alimentar e nutricional das crianças brasileiras, esse estudo propõe uma avaliação econômica do cardápio da alimentação escolar em um município do Sul do Brasil. Foi aplicada a razão custo-efetividade (RCE), a fim de relacionar o custo monetário de cada dia de alimentação com os benefícios nutricionais, estimados por meio da aplicação do Índice de Qualidade da Refeição. Na análise estatística, foram utilizados os testes de Mann-Whitney e o coeficiente de correlação de Spearman (valor de p = 0,05). O cardápio avaliado foi composto por 191 dias de alimentação, sendo 95 lanches e 96 refeições. Lanches (mediana = 0,11 [P25 = 0,09; P75 = 0,12]) apresentaram-se significativamente mais custo-efetivos na comparação com refeições (mediana = 0,08 [P25 = 0,06; P75 = 0,10]), o que representa uma vantagem à opção por refeições em vez de lanches, utilizando-se como critério estritamente objetivo a relação custo e qualidade nutricional. A aplicação da RCE como instrumento de decisão para gestão de cardápios em alimentação escolar foi positiva, uma vez que permite classificar a alimentação oferecida, em uma análise que combina economia e saúde.

## Abstract (en)

Considering the importance of the effective execution of the Brazilian National School Feeding Program in promoting food and nutritional security among Brazilian children, this study proposes an economic assessment of the school meal menu in a municipality in southern Brazil. The cost-effectiveness ratio was used to relate the monetary cost of meals to the nutritional benefits, which were estimated using the Meal Quality Index. The Mann-Whitney test and Spearman’s correlation coefficient (p-value = 0.05) were used for the statistical analysis. The menu consisted of 191 days of meals, including 95 snacks and 96 meals. Snacks (median = 0.11 [P25 = 0.09; P75 = 0.12]) were significantly more cost-effective compared to meals (median = 0.08 [P25 = 0.06; P75 = 0.10]), which constitutes an advantage in choosing meals over snacks, using the cost and nutritional quality relationship as a strictly objective criterion. The application of the cost-effectiveness ratio as a decision-making tool for menu management in the school meal program was positive, as it enables classifying the food offered in an analysis that combines economy and health.

## Abstract (es)

Dada la importancia de la ejecución efectiva del Programa Nacional de Alimentación Escolar con vistas a la promoción de la seguridad alimentaria y nutricional de los niños brasileños, este estudio propone una evaluación económica de la alimentación escolar en un municipio del sur de Brasil. Se aplicó la relación coste-efectividad (RCE) con el fin de relacionar el costo monetario de cada día de alimentación con los beneficios nutricionales, calculados mediante la aplicación del Índice de Calidad de la Comida. En el análisis estadístico se utilizaron las pruebas de Mann-Whitney y el coeficiente de correlación de Spearman (valor de p = 0,05). El menú evaluado constaba de 191 días de alimentación, con 95 refrigerios y 96 comidas. Los refrigerios (mediana = 0,11 [P25 = 0,09; P75 = 0,12]) resultaron significativamente más coste-eficaces en comparación con las comidas (mediana = 0,08 [P25 = 0,06; P75=0,10]), lo que representa una ventaja a favor de la opción de las comidas en lugar de los refrigerios, empleando como criterio estrictamente objetivo la relación entre el costo y la calidad nutricional. La aplicación de la RCE como instrumento de decisión para la gestión de menús en alimentación escolar fue positiva, puesto que permite clasificar la alimentación ofrecida, en un análisis que combina economía y salud.

## Introdução

A fome continua sendo um problema global na atualidade. A maioria dos países em desenvolvimento ainda está distante de alcançar o objetivo de acabar com a fome até 2030, uma das metas da Organização das Nações Unidas (ONU) para o desenvolvimento sustentável do planeta. Estima-se que entre 713 e 757 milhões de pessoas sofreram de fome em 2023 - aproximadamente 152 milhões a mais do que em 2019, antes da pandemia do coronavírus. A situação ainda parece se agravar com as crises econômicas que os governos enfrentam e desaceleração da economia mundial; com o desenrolar recente de guerras, como na Ucrânia e Faixa de Gaza; com a ocorrência cada vez mais frequente de catástrofes climáticas; além das variáveis subjacentes que historicamente contribuem para a persistência da iniquidade de acesso à alimentação ^1^.

Em acordo com 193 países, incluindo o Brasil, a iniciativa denominada Objetivos de Desenvolvimento Sustentável proposta pela ONU representa o comprometimento dos governos com um plano de ação global para proteger o planeta, promover sociedades pacíficas e inclusivas, oferecer educação universal de qualidade ao longo da vida dos indivíduos e erradicar a fome e a pobreza extrema. Por meio dessa iniciativa, os países concordaram em seguir a Agenda Pós-2015, considerada uma das mais ambiciosas da diplomacia internacional, a fim de atingir os 17 objetivos propostos até o ano de 2030 ^2^. Diante disso, é fundamental que as ações estatais sejam efetivas e que decisões alocativas de recursos garantam o emprego correto das verbas públicas a fim de se obter os melhores resultados e viabilizar o cumprimento das metas estabelecidas.

Programas de alimentação escolar constituem estratégias de promoção de segurança alimentar para crianças, assegurando o direito básico de acesso à alimentação adequada. No Brasil, o Programa Nacional de Alimentação Escolar (PNAE) é um dos mais abrangentes e complexos programas de alimentação escolar do mundo ^3^. O PNAE visa à transferência, em caráter suplementar, de recursos financeiros aos estados, Distrito Federal e aos municípios, para que sejam destinados a fim de suprir parcialmente as necessidades nutricionais dos alunos matriculados na rede pública de educação básica e entidades filantrópicas. O programa tem como objetivo contribuir para o crescimento e desenvolvimento biopsicossocial, rendimento escolar e formação de hábitos saudáveis dos alunos. Atualmente, é financiado com recursos do orçamento da União e gerenciado pelo Fundo Nacional de Desenvolvimento da Educação (FNDE) ^4^. Em razão de sua importância e complexidade, é recomendável que seja submetido à avaliação constante, a fim de verificar sua eficiência.

Avaliações econômicas tornam possível o aprimoramento de intervenções e justificam gastos públicos, uma vez que evidenciam o cumprimento - ou não - dos objetivos. Políticas públicas e programas governamentais devem estar em consonância com os princípios da economicidade - visando à minimização dos gastos públicos, sem comprometimento dos padrões de qualidade, garantindo sua eficiência e máximo retorno à sociedade ^5^.

Tratando-se de alimentação escolar, o nutricionista é o profissional responsável por sua gestão executiva, embasada por normativas específicas ^4^ ^,^ ^6^ ^,^ ^7^. Além disso, é dever do nutricionista membro do PNAE garantir que o planejamento do cardápio e a compra dos insumos sejam realizados com responsabilidade, assegurando que a destinação dos recursos públicos seja eficiente.

Diante da necessidade de encontrar a sustentabilidade econômica das operações e garantir o emprego correto das verbas públicas, é fundamental que se encontre respostas para questões básicas da rotina do gestor do PNAE. Além de conhecer o custo monetário e o valor nutricional das refeições do cardápio, é preciso relacionar essas variáveis para que se possa avaliar o custo-efetividade de cada escolha, e assim justificar a opção por um tipo de refeição em detrimento de outro. Dessa forma, o presente estudo propõe-se a avaliar economicamente a alimentação oferecida em escolas públicas do Município de Morro da Fumaça, Santa Catarina, durante o período compreendido entre fevereiro e dezembro de 2021, comparando os custos diretos de produção e os respectivos benefícios nutricionais de refeições e lanches que compuseram o cardápio.

## Materiais e métodos

### Delineamento do estudo

A pesquisa consiste em um estudo observacional analítico retrospectivo de avaliação econômica do tipo avaliação custo-efetividade, baseado na avaliação do cardápio oferecido aos alunos da rede municipal de Ensino Fundamental na cidade de Morro da Fumaça, durante um ano letivo. O horizonte de tempo da avaliação econômica refere-se ao período de um ano escolar, compreendido entre fevereiro e dezembro de 2021. A perspectiva da avaliação econômica do estudo foi baseada na ótica do pagador (ou seja, Prefeitura Municipal de Morro da Fumaça), identificando-se custos diretos relativos à produção da alimentação escolar e respectivos desfechos dietéticos representados pelo Índice de Qualidade da Refeição (IQR), descrito a seguir.

### Local da pesquisa

O Município de Morro da Fumaça está localizado ao sul do Estado de Santa Catarina, com população estimada de 18.537 habitantes (pequeno porte), segundo o Censo Populacional do Instituto Brasileiro de Geografia e Estatística (IBGE) de 2022 ^8^.

Ao longo de 2021, estavam matriculados no Ensino Fundamental 1.899 alunos, distribuídos entre sete escolas sob administração municipal de Morro da Fumaça. O cardápio oferecido foi igual nas escolas, sendo oferecida uma refeição por turno, no intervalo entre as aulas. Para as escolas que atendem nos turnos matutino e vespertino, o cardápio oferecido foi o mesmo em ambos os turnos.

### Fontes de dados

Os dados secundários utilizados no estudo foram coletados na Central de Alimentos do município, tendo sido coletados, transcritos e verificados por pares em uma única base de dados. As bases de dados municipais foram complementadas com informações de outras fontes, como o IBGE, para realização de análise de sensibilidade.

Todas as refeições do cardápio praticado no ano letivo de 2021 foram analisadas. No caso de refeições planejadas com mais de uma opção de preparação, ambas foram incluídas na análise, calculando-se uma média de custo e valor nutricional das duas preparações. Os cardápios para alunos que seguem dietas especiais (n = 61) não foram incluídos no estudo.

Foram analisadas as fichas técnicas de preparação dos itens que integravam o cardápio. Nas fichas técnicas, foram utilizados os valores *per capita* reais de alimentos e preparações servidos aos estudantes, a partir das quantidades dos itens alimentares adquiridos e destinados ao programa escolar ao nível de Ensino Fundamental no ano de 2021. Os dados constavam em Autorizações de Fornecimento - documento oficial do município - onde eram apresentados dados como a descrição dos itens alimentares e respectivas unidades de medida, quantidades compradas e preço. Os itens alimentares adquiridos foram distribuídos pelas refeições planejadas nos cardápios ofertados ao longo do ano e divididos pelo número total de alunos matriculados no Ensino Fundamental em 2021. Os valores obtidos ainda foram submetidos à comparação com valores de referência definidos pela *Instrução Normativa nº 1.568/2021* da Secretaria Estadual de Educação de Santa Catarina ^9^. Optou-se por utilizar o *per capita* referido na Instrução Normativa para 40 itens que apresentaram discrepância na comparação.

### Análise nutricional

Itens que apresentavam alguma particularidade para o estabelecimento da quantidade per capita do alimento tiveram metodologias específicas de cálculo. Alimentos adquiridos em unidades foram convertidos em peso (em quilogramas, Kg) ou volume (em litros, L), utilizando como referência dados da Companhia de Entrepostos e Armazéns Gerais de São Paulo (CEAGESP) ^10^.

O consumo de sal *per capita* foi contabilizado a partir do cômputo do sal incluído no preparo dos itens do cardápio. Saladas servidas cruas foram consideradas sem uso de sal no preparo. O uso de vinagre foi contabilizado em refeições nas quais foram ofertadas saladas no cardápio. A porção *per capita* de frutas foi calculada a partir do volume adquirido para uso na alimentação escolar em relação à oferta de preparações contendo frutas nos cardápios das escolas, utilizando-se porções usuais de consumo de cada tipo de fruta ^11^, uma vez que não constava a variedade oferecida no cardápio, apenas o nome genérico “fruta”. No serviço, os funcionários têm autonomia para utilizar a variedade mais própria para o consumo naquele momento, considerando a sazonalidade e o grau de maturação das frutas adquiridas. O Quadro 1 apresenta os valores *per capita* utilizados para frutas nos cardápios.

O cálculo do custo das preparações foi baseado na utilização do preço por quilograma de cada item alimentício adquirido. Itens adquiridos em unidades ou pacotes foram convertidos em peso (Kg) ou volume (L) correspondentes para cálculo do preço por unidade de medida padronizada. Itens que apresentavam variação de preços ao longo do ano tiveram preço médio calculado a partir das aquisições realizadas ao nível de governo municipal.

**Quadro 1:** Descrição dos valores utilizados para cálculo do *per capita* de frutas.

FRUTA	QUANTIDADE TOTAL ADQUIRIDA (KG)	FATOR DE CORREÇÃO	QUANTIDADE TOTAL CORRIGIDA (KG)	PORÇÃO (KG)	MEDIDA CASEIRA	PORÇÕES SERVIDAS	DIAS
Abacate	100,0	1,51	66,23	0,05	1 colher (sopa) cheia, picado	1.899	1
Abacaxi	2.559,4	1,93	1.326,11	0,07	1/2 fatia média	35.363	19
Banana	5.444,0	1,66	3.279,52	0,04	1/3 unidade média	140.551	74
Caqui chocolate	250,0	1,06	235,85	0,07	1/2 unidade média	3.798	2
Laranja	2.500,0	1,46	1.712,33	0,13	1/2 unidade média	19.026	10
Laranja poncã	940,0	1,36	691,18	0,09	1/2 unidade média	10.240	5
Maçã	2.627,0	1,24	2.118,55	0,09	1/2 unidade média	28.247	15
Mamão	600,0	1,38	434,78	0,08	1/3 fatia média	7.673	4
Manga	150,0	1,61	93,17	0,11	1/2 unidade média	1.331	1
Maracujá	250,0	2,16	115,74	0,05	1/2 unidade média	5.144	3
Melancia	4.450,0	1,90	2.342,11	0,13	1/3 fatia média	35.132	19
Morango	90,0	1,12	80,36	0,05	5 unidades médias	1.889	1
Pitaia	320,0	2,16	148,15	0,05	1/2 unidade média	6.584	3

Fonte: elaboração própria.

A lista de valores de fatores de correção e cocção utilizados nas fichas técnicas foi obtida a partir do manual do PNAE ^12^ e de Ornellas ^13^. Não foi encontrada referência de fator de correção para a pitaia na literatura, portanto, optou-se por utilizar o mesmo valor da fruta maracujá, uma vez que são estruturalmente semelhantes.

O cálculo do valor nutricional das refeições foi baseado na composição centesimal dos alimentos extraída da *Tabela Brasileira de Composição de Alimentos* (TACO) ^14^. Alimentos cuja composição nutricional não constava na TACO foram acessados a partir da *Tabela Brasileira de Composição de Alimentos* (TBCA) ^15^. Foram calculados: valor energético total, percentual calórico proveniente de carboidratos, proteínas e lipídios, ácidos graxos saturados, ácidos graxos insaturados, quantidade de sódio, ferro, cálcio, fibras alimentares, vitamina A e vitamina C de todas as refeições do cardápio.

### Desfechos dietéticos

O presente estudo considerou desfechos dietéticos para avaliação da adequação dos cardápios em relação aos custos para cada dia letivo na rede municipal de ensino. Os desfechos dietéticos foram avaliados por métricas propostas para cálculo do IQR. Desenvolvido por Bandoni ^16^, o indicador é recomendado para avaliação global de cardápios, a partir de avaliação objetiva da adequação no consumo de determinados macros e micronutrientes, assim como atendimento de recomendações dietéticas relativas ao consumo de determinados grupos alimentares, ingredientes e diversidade na alimentação.

A aplicação do IQR foi baseada na síntese da pontuação nutricional de cada cardápio a partir de dez variáveis analisadas: percentual calórico proveniente de carboidratos, proteínas e lipídios, ácidos graxos saturados, ácidos graxos insaturados, quantidade de sódio (mg), quantidade de colesterol (mg), adequação na oferta de frutas, verduras e legumes (g), adequação na oferta de açúcares livres e variabilidade do cardápio. Os pontos de corte estabelecidos para pontuação do IQR consideraram a população atendida pelo serviço, sendo baseados nas recomendações do PNAE ^17^ para escolares matriculados no Ensino Fundamental em período parcial. Assim, a ferramenta foi adaptada para considerar a oferta de 20% da necessidade nutricional diária dos alunos. Tendo em vista que algumas variáveis do IQR não estavam contempladas na legislação do PNAE, optou-se por usar as recomendações da Organização Mundial da Saúde (OMS)/Organização das Nações Unidas para a Alimentação e a Agricultura (FAO) ^18^. No Quadro 2, estão apresentadas as variáveis que compuseram a pontuação do IQR, pontos de corte, intervalos considerados e as respectivas pontuações, além da referência utilizada. Para o estabelecimento da pontuação do IQR, foi considerada uma margem de 10% entre o valor da referência e o valor ofertado pela refeição ou lanche, a fim de que pequenas variações do valor de referência também pudessem ser consideradas para a pontuação no índice. Dessa forma, a pontuação final do IQR para cada refeição analisada poderia variar entre 0 e 100, sendo 100 o melhor desempenho possível. A classificação das refeições conforme desempenho no IQR utilizou os mesmos critérios adotados por Bandoni ^16^, sendo considerada refeição adequada aquela que obteve escore acima de 80, refeição que necessita de melhoras aquela com escore entre 51 e 80 e, refeição inadequada, a que obteve escore inferior a 50.

**Quadro 2:** Descrição das variáveis que compõem o Índice de Qualidade da Refeição (IQR).

VARIÁVEL	RECOMENDAÇÃO	MARGEM (10% DA RECOMENDAÇÃO) *	REFERÊNCIA
Adequação na oferta de frutas, verduras e legumes	56g	Escore 10: 56g ou mais; Escore 5: entre 50,4-55,9g	PNAE
Oferta de proteína	10 a 15% do VET	Escore 10: entre 10-15%; Escore 5: entre 9-10% ou 15,1-16%	PNAE
Oferta de carboidrato	55 a 65% do VET	Escore 10: entre 55-65%; Escore 5: entre 49,5-54,9% ou 65,1-70,5%	PNAE
Oferta de gorduras totais	15 a 30% do VET	Escore 10: entre 15-30%; Escore 5: entre 13,5-14,9% ou 30,1-31,5%	PNAE
Oferta de gordura saturada	≤ 7% do VET	Escore 10: 7% ou menos; Escore 5: entre 7,1-7,7%	PNAE
Oferta de gordura insaturada	6 a 10% do VET	Escore 10: entre 6-10%; Escore 5: entre 5,4-5,9% ou 10,1-10,6%	OMS
Colesterol	≤ 60mg	Escore 10: 60mg ou menos; Escore 5: entre 60-66mg	OMS
Adequação na oferta de açúcares livres	≤ 7% do VET	Escore 10: até 7%; Escore 5: entre 7,1-7,7%	PNAE
Adequação na oferta de sódio	≤ 600mg	Escore 10: até 600mg; Escore 5: entre 601-660 mg	PNAE
Variabilidade do cardápio		Número de alimentos diferentes: - Escore 7: 11 ou mais; Escore 0: entre 1-4 Número de grupos alimentares diferentes: - Escore 3: 5 ou mais; Escore 0: entre 1-2	IQR **

OMS: Organização Mundial da Saúde; PNAE: Programa Nacional de Alimentação Escolar; VET: valor energético total.

Fonte: elaboração própria.

* Valores diferentes dos mencionados no quadro recebem escore 0;

** Metodologia desenvolvida por Bandoni ^16^.

### Estimativa de custos

Os custos no contexto da alimentação escolar no Município de Morro da Fumaça foram estimados por meio do cálculo de custos diretos relativos aos alimentos utilizados como ingredientes nas preparações servidas no cardápio escolar ao longo do ano de 2021. A partir dos dados das Autorizações de Fornecimento, foram estimados preços médios pagos por unidade de cada ingrediente adquirido pela Prefeitura Municipal ao longo do ano. O preço médio por unidade foi convertido em preço por unidade de medida padronizada de peso (Kg) ou volume (L).

Em seguida, foram aplicados fatores de correção e cocção para identificação do custo direto do ingrediente correspondente à porção *per capita* da parte comestível do alimento preparada pronta para consumo, conforme porção do alimento descrita na ficha técnica da preparação servida aos alunos da rede municipal de ensino (em gramas *per capita*). A soma dos custos diretos dos ingredientes de cada cardápio resultou no custo direto das preparações servidas a cada aluno (em Reais por dia letivo).

### Análise custo-efetividade

As comparações entre alternativas de cardápios da alimentação escolar foram baseadas na classificação de cada preparação como lanche ou refeição. Os cardápios cuja fonte principal de carboidrato foi pão, bolo, bolacha ou rosca, geralmente acompanhado por café, leite ou suco integral e uma porção de fruta, foram classificados como lanches. Os cardápios compostos por preparações que envolvem cocção e apresentam produção mais elaborada, consequentemente necessitando de maior quantidade de mão de obra e mais tempo para o preparo, como arroz, feijão, macarrão, carnes, acompanhados de saladas e uma porção de fruta como sobremesa, foram classificados como refeições. De uma maneira geral, o cardápio foi composto por lanches nas segundas e sextas-feiras e por refeições nas terças, quartas e quintas-feiras, a fim de facilitar a logística da operação.

A avaliação econômica foi realizada pelo cálculo da razão custo-efetividade (RCE), que sintetiza os resultados da comparação de diferentes estratégias de cuidado em saúde, a partir da identificação do desfecho de uma alternativa (denominador) em relação ao custo (numerador). O indicador representa o custo por unidade de efetividade da alternativa sob análise (custo/efetividade). Além da RCE, foi estimada a razão custo-efetividade incremental (RCEI), a partir da seleção da alternativa de menor RCE como cardápio de base para comparação das demais alternativas de alimentação escolar. O cálculo da RCEI é baseado na divisão da diferença entre custo da alternativa em avaliação em relação à base de comparação (numerador) pela diferença entre desfechos entre alternativa em avaliação e base de comparação (denominador), expressa pela Equação 1.

$$RCEI=\frac{C_{n}-C_{b}}{D_{n}-D_{b}}$$ (1)

Sendo que *C*_*n*_ é o custo da alternativa de cardápio em avaliação; *C*_*b*_ é o custo da base de comparação; *D*_*n*_ é o desfecho da alternativa de cardápio em avaliação; *D*_*b*_ é o desfecho da base de comparação (no caso, cardápio de menor RCE ao longo do ano de 2021).

Em seguida, foi realizada análise de sensibilidade probabilística multivariada dos resultados de RCEI para verificação da robustez dos resultados da avaliação econômica. A análise de sensibilidade refere-se ao procedimento de identificação de parâmetros-chave da avaliação econômica que sejam sujeitos a flutuações, potencialmente alterando custos e/ou desfechos de uma, ou mais alternativas sob avaliação e modificando a seleção da alternativa com melhor RCE.

Dentro do contexto da presente avaliação da alimentação escolar em Morro da Fumaça, identificaram-se como parâmetros-chave preços e pesos das porções dos alimentos componentes das preparações nos cardápios da alimentação escolar em Morro da Fumaça. Assim, a análise de sensibilidade probabilística multivariada baseou-se na estimativa de cenários mediante aplicação de modificações nos preços e porções de alimentos em cada cardápio sob avaliação.

As mudanças de preços dos alimentos foram baseadas em aplicação de simulação de Monte Carlo, utilizando-se distribuição de probabilidade fundamentada no padrão de ocorrência de variações mensais dos preços reais para cada alimento registradas no Índice Nacional de Preços ao Consumidor Amplo (IPCA), disponibilizado pelo IBGE para Região Metropolitana de Porto Alegre no período de janeiro de 2018 a dezembro de 2024 (ou seja, três anos antes, durante e três anos depois do ano letivo de 2021) ^19^. A Região Metropolitana de Porto Alegre foi selecionada entre diferentes locais de pesquisa de preços do IPCA em decorrência da maior proximidade com Morro da Fumaça. Complementarmente, as variações nos pesos das porções de alimentos foram baseadas em sorteio aleatório de valores no intervalo de 90% a 110% do peso do alimento na ficha técnica padronizada das preparações da alimentação escolar do município.

A análise de sensibilidade foi fundamentada na combinação de alterações em parâmetros de preços e pesos das porções de múltiplos alimentos utilizados para preparação dos cardápios da alimentação escolar no município, gerando 1.000 cenários diferentes para cada dia alimentar, conforme Quadro 3.

**Quadro 3:** Parâmetros para análise de sensibilidade multivariada.

INGREDIENTE	PREÇO (R$/KG)	VARIAÇÃO IPCA-POA (2018-2024)		PORÇÃO (KG)	LIMIARES DE VARIAÇÃO	
		MÉDIA	DP		INFERIOR	SUPERIOR
Abacate	7.100	1.208	4.804	0,053	-10%	+10%
Abacaxi	1.970	0.951	5.839	0,071	-10%	+10%
Açafrão	94.000	0.456	1.057	0,002	-10%	+10%
Aipim descascado	5.050	-0.648	7.108	0,035	-10%	+10%
Alface	6.280	1.507	9.592	0,020	-10%	+10%
Alho	22.800	0.456	1.057	0,002	-10%	+10%
Arroz	4.360	0.922	3.981	0,045	-10%	+10%
Arroz (1/2 porção)	2.180	0.922	3.981	0,023	-10%	+10%
Atum	59.160	0.423	1.925	0,006	-10%	+10%
Atum (1/2 porção)	29.580	0.423	1.925	0,003	-10%	+10%
Banana	3.310	0.643	6.821	0,039	-10%	+10%
Banana (1/2 porção)	3.310	0.643	6.821	0,039	-10%	+10%
Batata-doce	4.720	1.629	12.939	0,045	-10%	+10%
Batata inglesa	4.720	1.629	12.939	0,045	-10%	+10%
Bebida láctea	2.500	0.648	1.785	0,165	-10%	+10%
Beterraba	2.700	1.337	10.680	0,030	-10%	+10%
Bolacha caseira	27.610	0.446	1.720	0,045	-10%	+10%
Bolo	18.500	0.440	2.648	0,076	-10%	+10%
Brócolis	9.100	1.130	9.647	0,030	-10%	+10%
Cacau em pó	41.700	0.557	1.369	0,018	-10%	+10%
Cacau em pó (1/2 porção)	20.850	0.557	1.369	0,009	-10%	+10%
Café	17.060	0.542	2.718	0,012	-10%	+10%
Café (1/2 porção)	8.530	0.542	2.718	0,006	-10%	+10%
Canela em pó	118.400	0.456	1.057	0,008	-10%	+10%
Caqui chocolate	4.500	1.208	4.804	0,066	-10%	+10%
Carne em cubos	31.200	0.721	3.674	0,070	-10%	+10%
Carne moída	31.520	0.721	3.674	0,080	-10%	+10%
Cebola	3.850	2.462	15.603	0,004	-10%	+10%
Cenoura	3.140	0.885	14.143	0,045	-10%	+10%
Cereal	10.470	0.813	3.216	0,105	-10%	+10%
Chuchu	3.540	1.233	7.819	0,030	-10%	+10%
Colorau	7.370	0.456	1.057	0,002	-10%	+10%
Cominho em pó	99.000	0.456	1.057	0,002	-10%	+10%
Couve	11.510	1.233	7.819	0,015	-10%	+10%
Couve-flor	5.050	1.154	11.524	0,032	-10%	+10%
Ervilha	4.800	0.392	1.957	0,241	-10%	+10%
Farinha de mandioca/milho	4.700	0.526	1.543	0,017	-10%	+10%
Feijão	8.300	0.614	4.431	0,021	-10%	+10%
Feijão (1/2 porção)	4.150	0.614	4.431	0,011	-10%	+10%
Frango (coxa e sobrecoxa)	8.020	0.633	2.038	0,060	-10%	+10%
Frango (tipo sassami)	8.450	0.633	2.038	0,060	-10%	+10%
Frango (1/2 porção)	4.225	0.633	2.038	0,030	-10%	+10%
Laranja	3.240	1.709	10.200	0,158	-10%	+10%
Laranja poncã	3.040	2.885	8.860	0,092	-10%	+10%
Leite	3.130	0.914	6.683	0,165	-10%	+10%
Leite (1/2 porção)	3.130	0.914	6.683	0,083	-10%	+10%
Limão	4.000	1.208	4.804	0,006	-10%	+10%
Lombo suíno	20.220	0.633	2.889	0,058	-10%	+10%
Louro	46.250	0.456	1.057	0,000	-10%	+10%
Maçã	6.210	1.266	5.418	0,093	-10%	+10%
Macarrão	4.070	0.481	2.421	0,055	-10%	+10%
Macarrão (1/2 porção)	2.035	0.481	2.421	0,028	-10%	+10%
Mamão	4.050	1.249	11.011	0,092	-10%	+10%
Manga	3.790	1.100	9.472	0,109	-10%	+10%
Manteiga	23.000	0.678	3.814	0,020	-10%	+10%
Manteiga (1/2 porção)	11.500	0.678	3.814	0,010	-10%	+10%
Maracujá	5.290	1.208	4.804	0,049	-10%	+10%
Melancia	1.590	1.208	4.804	0,145	-10%	+10%
Milho-verde	4.800	0.788	2.164	0,724	-10%	+10%
Milho-verde (1/2 porção)	4.800	0.788	2.164	0,241	-10%	+10%
Moranga	1.780	1.337	10.680	0,035	-10%	+10%
Moranga (1/2 porção)	0.890	1.337	10.680	0,018	-10%	+10%
Morango	14.500	1.751	11.366	0,048	-10%	+10%
Óleo	9.920	0.603	4.917	0,003	-10%	+10%
Orégano	198.000	0.456	4.917	0,000	-10%	+10%
Ovos	8.860	0.596	3.660	0,055	-10%	+10%
Pão caseiro de aipim	20.060	0.479	1.734	0,038	-10%	+10%
Pão d’água	10.400	0.479	1.734	0,038	-10%	+10%
Pão de sanduíche	10.400	0.361	2.567	0,038	-10%	+10%
Pão doce	10.400	0.579	2.271	0,038	-10%	+10%
Pão doce (1/2 porção)	5.200	0.579	2.271	0,019	-10%	+10%
Pão francês	10.400	0.479	1.734	0,038	-10%	+10%
Pão para cachorro-quente	10.400	0.361	2.567	0,038	-10%	+10%
Pitaia	6.900	1.208	4.804	0,049	-10%	+10%
Queijo muçarela	34.490	0.503	2.011	0,020	-10%	+10%
Queijo muçarela (1/2 porção)	17.245	0.503	2.011	0,010	-10%	+10%
Repolho	2.930	1.215	9.924	0,016	-10%	+10%
Rosca de polvilho	15.900	0.443	1.079	0,022	-10%	+10%
Sal	0.950	0.456	4.917	0,002	-10%	+10%
Suco de frutas	12.910	0.455	1.518	0,165	-10%	+10%
Tempero verde	14.330	1.432	5.020	0,006	-10%	+10%
Tomate	4.690	1.763	16.154	0,043	-10%	+10%
Tomate (1/2 porção)	2.345	1.763	16.154	0,022	-10%	+10%
Vinagre	4.260	0.638	1.596	0,001	-10%	+10%

DP: desvio padrão; IPCA-POA: Índice Nacional de Preços ao Consumidor Amplo para Região Metropolitana de Porto Alegre.

Fonte: elaboração própria.

Os resultados da análise de sensibilidade foram utilizados para composição do gráfico de dispersão para identificação da distribuição de cenários a partir de diferenciais de custos e desfechos, assim como estimativa de elasticidades dos componentes de custos e desfechos dos cardápios em relação à RCEI, a partir de regressão *log-linear* expressa na Equação 2.

$$logRCEI=\beta_{0}+\beta_{1}\times logC+\beta_{2}\times logD+\sigma$$ (2)

Sendo que *C* é a matriz de componentes de custo e *D* é a matriz de componentes de desfecho de cada cardápio da alimentação escolar no município. Os coeficientes *β*_*1*_ e *β*_*2*_ representam elasticidades (*e*) dos componentes de custos e desfechos, destacando a magnitude dos efeitos das alterações nos preços e porções de alimentos sobre RCEI dos cardápios em avaliação. O *σ* representa o termo de erro na equação. Os resultados da regressão também foram utilizados para a construção de gráfico de tornado com principais fatores de influência sobre RCEI.

A análise estatística foi executada utilizando-se o programa SPSS 25.0 (https://www.ibm.com/) e o nível de significância adotado foi 0,05. A normalidade foi verificada por meio da aplicação do teste de Kolmogorov-Smirnov. A descrição das variáveis foi realizada por meio de medianas e intervalos interquartis. A comparação entre lanches e refeições foi feita pela aplicação do teste de Mann-Whitney. A correlação entre custo e IQR para cada tipo de alimentação servida foi verificada pelo coeficiente de correlação de Spearman.

As questões éticas que envolvem a execução desse estudo estão resguardadas pela autorização para realização da pesquisa. A submissão ao Comitê de Ética em Pesquisa não foi necessária, visto que o estudo não envolveu seres humanos. O projeto (nº 447/2023) foi registrado na Comissão de Pesquisa (COMPESQ) da Universidade Federal de Ciências da Saúde de Porto Alegre (UFCSPA).

## Resultados

O cardápio avaliado foi composto por 191 dias de alimentação, sendo 95 lanches e 96 refeições. Os valores da mediana de custo e IQR e suas respectivas medidas de variação podem ser visualizados na Tabela 1. Lanches apresentaram-se significativamente mais caros, embora apresentassem também maior qualidade nutricional do que refeições dos cardápios avaliados.

A aplicação da RCE refletiu a vantagem nutricional associada ao custo de produção de cada dia de alimentação, mostrando-se refeições mais vantajosas na comparação com lanches. A mediana da RCE e sua respectiva variação estão apresentadas na Tabela 1.

**Tabela 1:** Características das preparações na alimentação escolar. Morro da Fumaça, Santa Catarina, Brasil, 2021.

Características das preparações	Total (n = 191)	Lanches (n = 95)	Refeições (n = 96)	Valor de p
	Mediana [P25; P75]	Mediana [P25; P75]	Mediana [P25; P75]	
Kcal	645,8 [597,8; 711,4]	681,1 [617,1; 718,2]	615,2 [375,6; 689,4]	< 0,001
Carboidratos (g)	120,4 [107,3; 131,6]	127,8 [116,6; 131,6]	107,3 [47,0; 120,6]	< 0,001
Proteínas (g)	18,3 [14,0; 23,3]	15,5 [13,3; 18,1]	21,7 [18,3; 25,9]	< 0,001
Lipídios (g)	12,8 [10,4; 15,7]	13,8 [10,0; 17,0]	11,4 [10,5; 13,4]	< 0,001
Gorduras saturadas (g)	3,6 [2,6; 4,7]	4,7 [3,9; 6,9]	3,0 [2,5; 3,5]	< 0,001
Gorduras insaturadas (g)	6,8 [5,9; 8,4]	6,1 [5,3; 7,7]	7,5 [6,2; 8,7]	0,001
Fibras alimentares (g)	16,9 [11,4; 20,6]	20,5 [15,8; 21,0]	12,1 [4,6; 18,3]	< 0,001
Sódio (mg)	840,1 [268,3; 1.668,9]	268,3 [132,3; 526,7]	1.660,1 [849,2; 2.416,5]	< 0,001
Ferro (mg)	2,9 [2,4; 3,2]	3,1 [2,8; 3,5]	2,6 [2,0; 3,2]	< 0,001
Vitamina A (μg)	135,8 [135,3; 158,7]	135,8 [135,3; 138,5]	144,4 [39,7; 176,6]	0,931
Vitamina C (mg)	199,6 [105,7; 206,8]	201,3 [198,7; 231,4]	106,1 [5,4; 202,1]	< 0,001
Colesterol (mg)	45,5 [19,4; 86,1]	19,4 [17,3; 60,1]	73,3 [45,5; 101,0]	< 0,001
Açúcar de adição (g)	0,0 [0,0; 2,3]	2,3 [0,0; 6,3]	0,0 [0,0; 0,0]	< 0,001
Escore FLV	10,0 [10,0; 10,0]	10,0 [10,0; 10,0]	10,0 [0,0; 10,0]	< 0,001
Escore PTN	5,0 [0,0; 10,0]	0,0 [0,0; 5,0]	5,0 [0,0; 10,0]	0,02
Escore CHO	0,0 [0,0; 5,0]	0,0 [0,0; 0,0]	0,0 [0,0; 5,0]	0,014
Escore LIP	10,0 [0,0; 10,0]	10,0 [0,0; 10,0]	10,0 [3,8; 10,0]	0,418
Escore G.SAT	10,0 [5,0; 10,0]	5,0 [0,0; 10,0]	10,0 [10,0; 10,0]	< 0,001
Escore G.INS	5,0 [0,0; 10,0]	10,0 [5,0; 10,0]	0,0 [0,0; 10,0]	< 0,001
Escore COL	10,0 [0,0; 10,0]	10,0 [5,0; 10,0]	0,0 [0,0; 10,0]	< 0,001
Escore AAD	10,0 [10,0; 10,0]	10,0 [10,0; 10,0]	10,0 [10,0; 10,0]	0,013
Escore Na	10,0 [0,0; 10,0]	10,0 [10,0; 10,0]	0,0 [0,0; 0,0]	< 0,001
Escore variedade de alimentos	7,0 [5,5; 7,0]	7,0 [7,0; 7,0]	5,5 [4,0; 7,0]	< 0,001
Escore variedade de grupos	3,0 [2,0; 3,0]	2,0 [0,0; 3,0]	3,0 [3,0; 3,0]	< 0,001
IQR	65,0 [49,0; 75,0]	70,0 [58,5; 77,0]	55,0 [47,0; 65,0]	< 0,001
Custo (R$)	6,1 [4,5; 7,8]	7,4 [6,5; 7,9]	4,5 [2,2; 5,9]	< 0,001
Custo/Kcal	0,010 [0,007; 0,012]	0,010 [0,010; 0,012]	0,008 [0,004; 0,010]	< 0,001
RCE	0,1 [0,1; 0,1]	0,1 [0,1; 0,1]	0,1 [0,1; 0,1]	< 0,001
RCEI	0,2 [0,0; 0,4]	0,2 [0,2; 0,4]	0,2 [-0,1; 0,4]	< 0,001
ΔCusto	5,2 [3,6; 6,9]	6,5 [5,7; 7]	3,6 [1,3; 5,1]	< 0,001
ΔIQR	13,0 [-3,0; 23,0]	18,0 [6,5; 25,0]	3,0 [-5,0; 13,0]	< 0,001

AAD: açúcar de adição; CHO: carboidratos; COL: colesterol; FLV: frutas, legumes e verduras; G.INS: gorduras insaturadas; G.SAT: gorduras saturadas; IQR: Índice de Qualidade da Refeição; Kcal: calorias; LIP: lipídios; Na: sódio; PTN: proteína; RCE: razão custo-efetividade; RCEI: razão custo-efetividade incremental.

Fonte: elaboração própria.

A composição nutricional de lanches e refeições encontra-se representada pela mediana e sua variação correspondente na Tabela 1. Lanches apresentaram maior quantidade de carboidratos, gorduras, fibras alimentares, ferro, vitamina C, gorduras saturadas e açúcares de adição do que refeições. Já as refeições mostraram-se significativamente mais ricas em proteínas e gorduras insaturadas do que lanches, porém apresentaram maior quantidade de sódio e colesterol na mesma comparação. Para retinol, não houve diferença significativa entre lanches e refeições.

**Figura 1:**
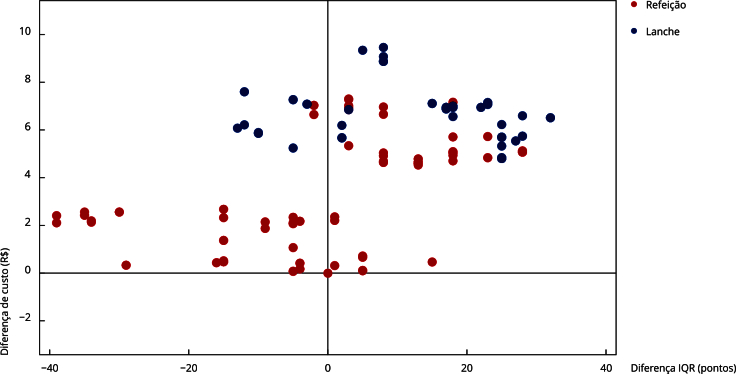
Análise de diferenciais de custos em relação a diferenciais de Índice de Qualidade da Refeição (IQR) nos cardápios do Programa Nacional de Alimentação Escolar (PNAE). Morro da Fumaça, Santa Catarina, Brasil, 2021.

A Figura 1 demonstra a dispersão dos diferenciais de custo e desfecho (IQR) para cada dia de alimentação que compôs o cardápio avaliado. Pode-se verificar que lanches apresentaram maior custo, mas também maior pontuação no IQR. Já as refeições apresentaram ampla variação de custo e IQR, demonstrando que cardápios com maior qualidade nutricional também eram mais caros.

**Figura 2:**
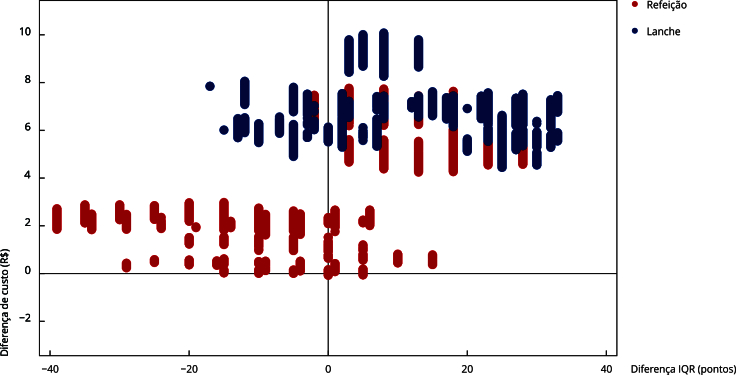
Análise de sensibilidade probabilística para diferenciais de custos em relação a diferenciais de Índice de Qualidade da Refeição (IQR) dos cardápios do Programa Nacional de Alimentação Escolar (PNAE). Morro da Fumaça, Santa Catarina, Brasil, 2021.

Embora o gráfico original de dispersão dos cardápios aponte inexistência de alternativas com menor custo em relação à base de comparação, os resultados da análise de sensibilidade probabilística multivariada apontam possibilidade de ocorrência de algumas alternativas de refeições com menor custo e maior IQR em comparação com cardápio selecionado como base de comparação (Figura 2).

A Tabela 2 apresenta a síntese das características dos cardápios obtidos a partir da análise de sensibilidade multivariada. É possível identificar substancial similaridade em relação ao padrão de características dos cardápios avaliados, entretanto, verifica-se maior amplitude na variabilidade de desfechos para refeições em comparação com lanches, assim como em termos de diferenciais de desfechos em relação ao cardápio de base de comparação.

**Tabela 2:** Características das preparações na alimentação escolar por análise de sensibilidade multivariada. Morro da Fumaça, Santa Catarina, Brasil, 2021.

Características das preparações	Total (n = 191)	Lanches (n = 95)	Refeições (n = 96)	Valor de p
	Mediana [P25; P75]	Mediana [P25; P75]	Mediana [P25; P75]	
Kcal	647,6 [593,0; 715,8]	677,3 [617,4; 722,1]	610,4 [371,2; 695,8]	< 0,001
Carboidratos (g)	119,7 [108,7; 130,7]	127,2 [117,6; 132,4]	109,3 [45,6; 121,7]	< 0,001
Proteínas (g)	17,8 [14,0; 23,6]	15,2 [13,3; 18,0]	21,8 [17,7; 26,9]	< 0,001
Lipídios (g)	12,7 [10,3; 15,8]	14,0 [10,2; 17,3]	11,5 [10,4; 13,4]	< 0,001
Gorduras saturadas (g)	3,6 [2,6; 4,8]	4,8 [3,8; 7,0]	3,0 [2,5; 3,5]	< 0,001
Gorduras insaturadas (g)	6,8 [5,7; 8,5]	6,3 [5,3; 7,7]	7,4 [6,2; 8,9]	< 0,001
Fibras alimentares (g)	17,1 [8,1; 20,5]	20,1 [15,9; 21,1]	11,9 [4,6; 18,3]	< 0,001
Sódio (mg)	863,7 [270,1; 1.654,4]	269,8 [132,4; 559,1]	1.648,3 [885,3; 2.298,0]	< 0,001
Ferro (mg)	3,0 [2,3; 3,3]	3,1 [2,8; 3,4]	2,6 [2,0; 3,3]	< 0,001
Vitamina A (μg)	138,1 [131,0; 159,9]	137,2 [133,8; 142,4]	146,0 [40,2; 176,3]	< 0,001
Vitamina C (mg)	199,8 [14,9; 209,3]	204,8 [197,8; 231,3]	100,5 [5,4; 202,4]	< 0,001
Colesterol (mg)	47,3 [20,1; 84,4]	20,0 [17; 57,6]	72,8 [46,4; 103,2]	< 0,001
Açúcar de adição (g)	0,0 [0,0; 2,5]	2,5 [0,0; 5,9]	0,0 [0,0; 0,0]	< 0,001
Escore FLV	10,0 [10,0; 10,0]	10,0 [10,0; 10,0]	10,0 [0,0; 10,0]	< 0,001
Escore PTN	5,0 [0,0; 10,0]	5,0 [0,0; 5,0]	5,0 [0,0; 10,0]	< 0,001
Escore CHO	5,0 [0,0; 5,0]	0,0 [0,0; 0,0]	5,0 [0,0; 5,0]	< 0,001
Escore LIP	10,0 [0,0; 10,0]	10,0 [0,0; 10,0]	10,0 [0,0; 10,0]	< 0,001
Escore G.SAT	10,0 [5,0; 10,0]	10,0 [0,0; 10,0]	10,0 [10,0; 10,0]	< 0,001
Escore G.INS	5,0 [0,0; 10,0]	10,0 [5,0; 10,0]	0,0 [0,0; 10,0]	< 0,001
Escore COL	10,0 [0,0; 10,0]	10,0 [10,0; 10,0]	0,0 [0,0; 10,0]	< 0,001
Escore AAD	10,0 [10,0; 10,0]	10,0 [10,0; 10,0]	10,0 [10,0; 10,0]	< 0,001
Escore Na	0,0 [0,0; 10,0]	10,0 [10,0; 10,0]	0,0 [0,0; 0,0]	< 0,001
Escore variedade de alimentos	7,0 [4,0; 7,0]	7,0 [7,0; 7,0]	5,5 [4,0; 7,0]	< 0,001
Escore variedade de grupos	3,0 [2,0; 3,0]	2,0 [0,0; 3,0]	3,0 [3,0; 3,0]	< 0,001
IQR	65,0 [50,0; 75,0]	70,0 [59,0; 77,0]	57,0 [43,0; 70,0]	< 0,001
Custo (R$)	6,1 [3,7; 7,7]	7,3 [6,5; 7,9]	4,5 [2,1; 5,9]	< 0,001
Custo/Kcal	0,010 [0,007; 0,012]	0,010 [0,01; 0,012]	0,008 [0,005; 0,01]	< 0,001
RCE	0,1 [0,1; 0,1]	0,1 [0,1; 0,1]	0,1 [0,1; 0,1]	< 0,001
RCEI	0,2 [0,0; 0,4]	0,3 [0,2; 0,5]	0,2 [-0,1; 0,3]	< 0,001
ΔCusto	5,3 [2,8; 6,8]	6,4 [5,6; 7,0]	3,6 [1,2; 5,1]	< 0,001
ΔIQR	13,0 [-2,0; 23,0]	18,0 [7,0; 25,0]	5,0 [-9,0; 18,0]	< 0,001

AAD: açúcar de adição; CHO: carboidratos; COL: colesterol; FLV: frutas, legumes e verduras; G.INS: gorduras insaturadas; G.SAT: gorduras saturadas; IQR: Índice de Qualidade da Refeição; Kcal: calorias; LIP: lipídios; Na: sódio; PTN: proteína; RCE: razão custo-efetividade; RCEI: razão custo-efetividade incremental.

Fonte: elaboração própria.

Na análise da correlação entre custo e IQR para lanches e refeições, obtida por meio do teste de correlação de Spearman, não se observou correlação significativa entre custo e IQR para lanches (rho = -0,016; p = 0,876). No entanto, para refeições, quanto maior o custo, maior a qualidade nutricional (rho = 0,218; p = 0,033).

Por fim, os resultados da regressão indicam maiores efeitos positivos e negativos decorrentes de mudanças nos custos dos alimentos componentes dos cardápios, em comparação com alterações nos componentes do IQR (Tabela 3).

**Tabela 3:** Coeficientes de elasticidade estimados por análise de sensibilidade multivariada de características das preparações na alimentação escolar. Morro da Fumaça, Santa Catarina, Brasil, 2021.

RCEI	e	EP	IC95%	Valor de p
Escore FLV	0,020	0,000	0,019; 0,021	< 0,001
Escore PTN	-0,032	0,000	-0,033; -0,032	< 0,001
Escore CHO	-0,046	0,000	-0,047; -0,046	< 0,001
Escore LIP	0,025	0,000	0,024; 0,026	< 0,001
Escore G.SAT	-0,040	0,000	-0,041; -0,040	< 0,001
Escore G.INS	-0,026	0,000	-0,027; -0,025	< 0,001
Escore COL	0,002	0,000	0,001; 0,003	< 0,001
Escore AAD	0,107	0,001	0,106; 0,109	< 0,001
Escore Na	-0,057	0,000	-0,058; -0,057	< 0,001
Escore variedade de alimentos	-0,002	0,000	-0,002; -0,001	0,001
Escore variedade de grupos	-0,063	0,000	-0,064; -0,062	< 0,001
Custo fruta	0,082	0,080	-0,074; 0,238	0,303
Custo pão d’água com carne moída	0,085	0,028	0,030; 0,141	0,003
Custo suco de uva	0,193	0,024	0,147; 0,240	< 0,001
Custo bolo	0,169	0,024	0,123; 0,215	< 0,001
Custo café com leite	0,203	0,030	0,144; 0,263	< 0,001
Custo bolacha caseira	-0,100	0,024	-0,147; -0,053	< 0,001
Custo vitamina de banana	0,541	0,036	0,471; 0,611	< 0,001
Custo rosca	0,048	0,024	0,001; 0,095	0,044
Custo sanduíche de frango com cenoura	0,344	0,040	0,265; 0,423	< 0,001
Custo pão com manteiga	0,217	0,033	0,153; 0,281	< 0,001
Custo vitamina de fruta	0,259	0,044	0,174; 0,345	< 0,001
Custo sanduíche queijo	0,103	0,033	0,038; 0,167	0,002
Custo risoto de frango	0,832	0,045	0,744; 0,921	< 0,001
Custo salada de alface	-0,019	0,024	-0,066; 0,027	0,411
Custo tomate	0,261	0,024	0,214; 0,308	< 0,001
Custo milho-verde cozido	0,200	0,024	0,153; 0,247	< 0,001
Custo carne em cubo refogada	0,082	0,025	0,033; 0,131	0,001
Custo cenoura ralada	0,039	0,024	-0,008; 0,086	0,106
Custo macarrão	0,211	0,027	0,157; 0,264	< 0,001
Custo salada de repolho	-0,042	0,024	-0,089; 0,006	0,084
Custo arroz	0,181	0,035	0,113; 0,250	< 0,001
Custo beterraba	0,108	0,024	0,061; 0,154	< 0,001
Custo carne moída	0,185	0,026	0,135; 0,236	< 0,001
Custo minestra com legumes	0,798	0,061	0,679; 0,918	< 0,001
Custo ovo cozido	0,263	0,024	0,216; 0,311	< 0,001
Custo salada de couve ou repolho	0,159	0,029	0,102; 0,216	< 0,001
Custo salada de batata mista	-0,034	0,034	-0,101; 0,033	0,321
Custo macarrão com carne moída	0,056	0,028	0,002; 0,110	0,042
Custo sanduíche de frango com tomate	-0,025	0,040	-0,103; 0,054	0,535
Custo pão com queijo	0,245	0,032	0,183; 0,307	< 0,001
Custo sopa de frango com arroz e legumes	0,687	0,054	0,581; 0,792	< 0,001
Custo purê de aipim	0,360	0,033	0,295; 0,426	< 0,001
Custo coxa/sobrecoxa de frango assada	0,154	0,029	0,096; 0,211	< 0,001
Custo feijão	0,278	0,035	0,210; 0,347	< 0,001
Custo canja de frango	0,406	0,043	0,321; 0,491	< 0,001
Custo sanduíche de queijo e tomate	0,576	0,037	0,503; 0,648	< 0,001
Custo omelete	-0,031	0,025	-0,080; 0,019	0,226
Custo salada de couve	0,016	0,025	-0,032; 0,064	0,523
Custo carne suína em cubos	0,078	0,027	0,026; 0,130	0,003
Custo pão doce ou bolo	-0,116	0,027	-0,169; -0,063	< 0,001
Custo sopa de frango com legumes	0,246	0,051	0,146; 0,347	< 0,001
Custo frango refogado	-0,015	0,029	-0,073; 0,043	0,611
Custo salada de brócolis ou couve-flor	-0,230	0,033	-0,295; -0,166	< 0,001
Custo salada de brócolis	-0,030	0,025	-0,078; 0,018	0,219
Custo farofa com cenoura	0,423	0,044	0,337; 0,509	< 0,001
Custo leite com cacau	0,108	0,033	0,043; 0,173	0,001
Custo carne refogada com cenoura	-0,030	0,026	-0,082; 0,022	0,256
Custo farofa com legumes	0,454	0,050	0,356; 0,552	< 0,001
Custo galinha ensopada com macarrão	0,168	0,036	0,097; 0,239	< 0,001
Custo arroz carreteiro	0,093	0,031	0,032; 0,155	0,003
Custo aipim	0,033	0,024	-0,013; 0,080	0,157
Custo sanduíche de frango ou queijo	0,167	0,040	0,088; 0,245	< 0,001
Custo farofa com couve	0,028	0,044	-0,058; 0,113	0,524
Custo salada de couve-flor	0,169	0,025	0,120; 0,217	< 0,001
Custo chuchu cozido	0,174	0,025	0,126; 0,223	< 0,001
Custo pão com queijo e tomate	-0,099	0,038	-0,173; -0,025	0,009
Custo arroz com carne moída	0,015	0,029	-0,042; 0,072	0,605
Custo ovos mexidos	0,083	0,026	0,033; 0,133	0,001
Custo salada de alface ou couve	0,038	0,034	-0,029; 0,104	0,268
Custo carne moída com legumes	-0,010	0,027	-0,064; 0,043	0,708
Custo carne em cubos com legumes	0,120	0,028	0,065; 0,175	< 0,001
Custo sanduíche de queijo com cenoura	0,507	0,036	0,436; 0,578	< 0,001
Custo café com leite ou leite com cacau	0,307	0,032	0,245; 0,369	< 0,001
Custo legumes	-0,252	0,041	-0,333; -0,172	< 0,001
Custo carne em cubos com molho	0,109	0,028	0,054; 0,163	< 0,001
Custo iogurte com cereal	0,142	0,031	0,081; 0,202	< 0,001
Custo arroz com galinha, milho e ervilha	0,364	0,046	0,273; 0,455	< 0,001
Custo macarrão com molho e atum	0,452	0,039	0,375; 0,529	< 0,001
Custo sanduíche de atum ou frango	0,532	0,039	0,456; 0,609	< 0,001

AAD: açúcar de adição; CHO: carboidratos; COL: colesterol; e: elasticidade; EP: erro padrão; FLV: frutas, legumes e verduras; G.INS: gorduras insaturadas; G.SAT: gorduras saturadas; IC95%: intervalo de 95% de confiança; LIP: lipídios; Na: sódio; PTN: proteína; RCEI: razão custo-efetividade incremental.

Fonte: elaboração própria.

**Figura 3:**
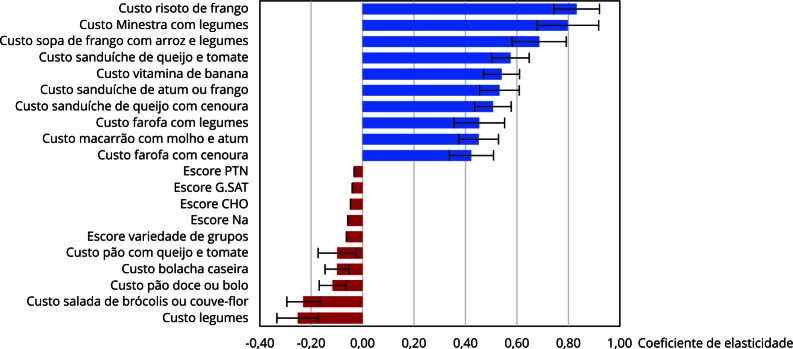
Coeficientes de elasticidade dos componentes de custo e qualidade nutricional dos cardápios do Programa Nacional de Alimentação Escolar (PNAE) em relação à razão custo-efetividade incremental (RCEI). Morro da Fumaça, Santa Catarina, Brasil, 2021.

A Figura 3 destaca dez fatores com maior elasticidade positiva e dez fatores com maior elasticidade negativa em relação à RCEI, incluindo respectivos intervalos de 95% de confiança (IC95%), indicando maiores efeitos positivos decorrentes de preparações com alimentos de origem animal.

CHO: carboidratos; G.SAT: gorduras saturadas; Na: sódio; PTN: proteína.

## Discussão

A aplicação da RCE como instrumento de decisão para gestão de cardápios na alimentação escolar mostrou-se positiva, uma vez que permitiu a classificação das refeições, considerando custo e benefícios nutricionais em uma análise que combina economia e saúde. A objetividade da avaliação e a facilidade de aplicação são características que justificam seu uso no planejamento da produção em unidades de alimentação e nutrição escolares (UANE). A utilização do IQR adaptado à população avaliada permitiu a avaliação objetiva e global do cardápio. Sarti et al. ^20^, avaliando cozinhas comunitárias em uma análise de custo-efetividade sobre políticas públicas de segurança alimentar e nutricional no Brasil, encontraram em seus resultados que a presença de nutricionista estava entre as variáveis de maior influência em melhores valores de RCE, além de apresentar significativa influência no nível de higiene das instalações e no conteúdo calórico das refeições ofertadas. Esses achados podem sugerir um benefício do uso da técnica de avaliação de tecnologias em saúde na rotina do nutricionista de UANEs, em favor da comunidade. Esses achados podem sugerir um benefício do uso da técnica de avaliação de tecnologias em saúde na rotina do nutricionista de UANEs, em favor da comunidade. Machado & Simões ^21^, em uma avaliação econômica semelhante, puderam identificar a melhor opção de café da manhã no serviço analisado, utilizando critérios objetivos considerando custos de produção e benefícios à saúde, e recomendaram a análise como base fundamental do processo de compra em instituições públicas.

Nesse estudo, a avaliação realizada nos cardápios do PNAE no Município de Morro da Fumaça indicou que lanches são mais custo-efetivos do que refeições, o que reflete uma vantagem das refeições sobre os lanches nessa comparação. Cabe destacar que nessa análise foram considerados somente os gastos com insumos alimentares no cálculo do custo das refeições. Uma análise mais complexa, que inclua no cálculo os custos com funcionários, gastos com água, luz e gás, pode apresentar resultados diferentes. Importante salientar ainda que, de acordo com os resultados encontrados por meio da aplicação do IQR, o cardápio apresentou necessidade de melhorias para a maioria das refeições avaliadas, mesmo em um município onde é realizada uma robusta complementação da verba repassada pelo FNDE para o custeio da alimentação escolar. Sabe-se que essa condição financeira não é realidade em todo o país e, estudos conduzidos em regiões com recursos municipais ou estaduais mais escassos e, consequentemente, com cardápio escolar mais limitado, podem eventualmente encontrar resultados ainda menos satisfatórios.

Em relação à composição nutricional de lanches e refeições, o cardápio analisado apresentou algumas peculiaridades. Lanches apresentaram maior quantidade de carboidratos, gorduras saturadas e açúcares de adição do que refeições. Ao contrário do que era esperado, também apresentaram maior quantidade de fibras alimentares, ferro e vitamina C, uma vez que contêm uma porção de fruta em sua composição. Já as refeições mostraram-se significativamente mais ricas em proteínas e gorduras insaturadas que lanches, porém, apresentaram maior quantidade de sódio e colesterol na mesma comparação. Para retinol, não houve diferença significativa entre lanches e refeições.

Além das questões de custo e qualidade nutricional, existem outras diferenças entre lanches e refeições que devem ser consideradas no momento da escolha da composição do cardápio. Questões operacionais, como tempo para pré-preparo, disponibilidade de mão de obra e infraestrutura da cozinha, também interferem na escolha entre lanche ou refeição. Gelli et al. ^22^ avaliaram custos e custo-efetividade de programas de alimentação escolar em países com população em situação de insegurança alimentar. Nessa pesquisa, os custos com refeições quentes corresponderam à metade do orçamento destinado à alimentação de escolares.

Verly-Junior et al. ^23^ analisaram a viabilidade de adequação de cardápios do PNAE em relação ao cumprimento das exigências nutricionais estabelecidas pelo programa. Os resultados demonstraram que é pouco provável a oferta de um cardápio que contemple todos os componentes nas quantidades exigidas, considerando os recursos disponíveis para compras de alimentos, e concluem que possivelmente grande parte dos cardápios praticados nas escolas não consiga cumprir a legislação do PNAE.

Pedraza et al. ^24^, em uma pesquisa de revisão da literatura sobre estudos avaliativos do PNAE, não incluíram em sua amostra estudos que considerassem custos e benefícios nutricionais em suas análises, possivelmente pela falta de pesquisas com essa característica. Nesse artigo, os resultados encontrados demonstraram que a execução do PNAE está aquém do recomendado em se tratando da oferta de refeições saudáveis. Observou-se que os cardápios analisados não ofertavam o percentual mínimo recomendado para suprir as necessidades nutricionais dos escolares, além de apresentarem baixa quantidade de frutas e hortaliças em sua composição.

O presente estudo utilizou dados *per capita* reais do serviço de alimentação escolar do município, o que pode ser considerado um ponto forte dessa pesquisa, por demonstrar a realidade, o que de fato foi comprado e destinado para alimentação dos escolares. A partir dos dados avaliados, identificou-se cumprimento parcial dos parâmetros estipulados pelo PNAE no cardápio do Ensino Fundamental no município.

Algumas limitações também foram observadas no desenvolvimento do estudo, como a ausência de padronização das porções *per capita* de alguns itens no cardápio do PNAE ao nível municipal. Por exemplo, no caso das frutas, os valores *per capita* foram estimados, uma vez que o dado sobre qual variedade foi ofertada em cada dia não estava disponível. Essa conduta é justificada pela autonomia dos zeladores escolares na escolha da variedade a ser oferecida, considerando o grau de maturação do alimento e a disponibilidade em cada época do ano, o que pode ser considerado uma vantagem prática do serviço para evitar ou reduzir o desperdício. Os valores de quantidades *per capita* de alguns temperos também estavam indisponíveis nos registros dos cardápios, potencialmente resultando em subestimação ou superestimação do uso no contexto do PNAE.

Este estudo considerou dados do ano de 2021 e analisou os preços pagos pelos alimentos durante esse ano. Assim, os preços utilizados representam um cenário interessante por repercutirem os preços vigentes durante a retomada de atividades pós-pandemia do coronavírus. Ademais, considerando a necessidade de aplicação de análise de sensibilidade sobre parâmetros-chave da avaliação econômica, foram utilizados dados de flutuações mensais de preços dos itens alimentares, a partir de distribuição de probabilidade capturada pelo IPCA referente à Região Metropolitana de Porto Alegre, devido à maior proximidade do Município de Morro da Fumaça. Os resultados da análise de sensibilidade apontaram que variações nos preços dos ingredientes componentes das preparações servidas na alimentação escolar apresentaram maiores efeitos positivos ou negativos sobre a RCEI do que componentes vinculados ao IQR, considerando variações no tamanho das porções servidas aos alunos.

Por fim, é importante destacar que a avaliação econômica conduzida no presente estudo utilizou o IQR como desfecho dietético da política pública de alimentação escolar no Município de Morro da Fumaça. Embora existam evidências robustas quanto aos efeitos de padrões de consumo alimentar sobre nutrição e saúde dos indivíduos ^18^ ^,^ ^25^ ^,^ ^26^ ^,^ ^27^ ^,^ ^28^ ^,^ ^29^ ^,^ ^30^ ^,^ ^31^, particularmente crianças e adolescentes em fase de crescimento e desenvolvimento ^32^ ^,^ ^33^ ^,^ ^34^ ^,^ ^35^, o IQR é baseado na identificação de consumo de marcadores de alimentação saudável ^16^.

Sendo assim, os efeitos em nutrição e saúde são identificáveis a curto, médio e longo prazo a partir de diferentes tipos de estudos longitudinais. A curto prazo, somente é possível a identificação de efeitos clínicos da dieta por meio de análise de marcadores bioquímicos (como glicemia, HDL, LDL, colesterol etc.). Em médio e longo prazo, torna-se possível a verificação de efeitos em dimensões antropométricas, clínicas e de saúde, como peso corporal, índice de massa corporal, risco para síndrome metabólica e outros marcadores bioquímicos (como hemoglobina glicada, HOMA-IR, entre outros), incluindo-se a redução da probabilidade de ocorrência de várias morbidades (p.ex.: diabetes tipo 2, hipertensão e doenças cardiovasculares). Consequentemente, a análise custo-efetividade conduzida no presente estudo, a partir de delineamento observacional retrospectivo com base em análise quantitativa de dados secundários de implementação da política pública, impede a observação direta de efeitos clínicos, nutricionais e de saúde relacionados ao IQR.

Entretanto, torna-se fundamental apontar que avaliações econômicas, em especial a análise custo-efetividade, medem a eficiência e a efetividade de alternativas de ação, sendo um instrumento de análise do valor para distintas intervenções sob comparação. Embora na tomada de decisão no planejamento de cardápios estejam envolvidos aspectos éticos, culturais, políticos, sociais e econômicos, a seleção de uma opção com melhor qualidade nutricional e menor custo torna-se uma questão de cunho científico e, simultaneamente, garante o melhor uso de recursos públicos para promoção do bem-estar social, portanto, deve ser avaliada. Dada a crescente necessidade de decisões em gestão pública serem baseadas em evidências e do emprego de recursos públicos considerar a eficiência e a efetividade das alternativas disponíveis, a disseminação dessa metodologia em pesquisa científica atende aos requisitos das melhores práticas de cuidados em saúde e gestão pública. Sendo assim, mais estudos devem ser conduzidos para que possam apoiar a prática dos gestores de unidades de alimentação e nutrição, particularmente no setor público, a partir de evidências científicas.
